# Altering cancer transcriptomes using epigenomic inhibitors

**DOI:** 10.1186/1756-8935-8-9

**Published:** 2015-02-24

**Authors:** Malaina Gaddis, Diana Gerrard, Seth Frietze, Peggy J Farnham

**Affiliations:** USC/Norris Comprehensive Cancer Center, University of Southern California, 1450 Biggy Street, NRT 6503, Los Angeles, CA 90089-9601 USA; School of Biological Sciences, University of Northern Colorado, Greeley, CO 80639 USA

**Keywords:** Epigenetic inhibitor, Histone acetylation, WNT signaling, C646, ICG-001, Colon cancer, Pancreatic cancer, TCF7L2, Cholesterol biosynthesis

## Abstract

**Background:**

Due to the hyper-activation of WNT signaling in a variety of cancer types, there has been a strong drive to develop pathway-specific inhibitors with the eventual goal of providing a chemotherapeutic antagonist of WNT signaling to cancer patients. A new category of drugs, called epigenetic inhibitors, are being developed that hold high promise for inhibition of the WNT pathway. The canonical WNT signaling pathway initiates when WNT ligands bind to receptors, causing the nuclear localization of the co-activator β-catenin (CTNNB1), which leads to an association of β-catenin with a member of the TCF transcription factor family at regulatory regions of WNT-responsive genes. The TCF/β-catenin complex then recruits CBP (CREBBP) or p300 (EP300), leading to histone acetylation and gene activation. A current model in the field is that CBP-driven expression of WNT target genes supports proliferation whereas p300-driven expression of WNT target genes supports differentiation. The small molecule inhibitor ICG-001 binds to CBP, but not to p300, and competitively inhibits the interaction of CBP with β-catenin. Upon treatment of cancer cells, this should reduce expression of CBP-regulated transcription, leading to reduced tumorigenicity and enhanced differentiation.

**Results:**

We have compared the genome-wide effects on the transcriptome after treatment with ICG-001 (the specific CBP inhibitor) versus C646, a compound that competes with acetyl-coA for the Lys-coA binding pocket of both CBP and p300. We found that both drugs cause large-scale changes in the transcriptome of HCT116 colon cancer cells and PANC1 pancreatic cancer cells and reverse some tumor-specific changes in gene expression. Interestingly, although the epigenetic inhibitors affect cell cycle pathways in both the colon and pancreatic cancer cell lines, the WNT signaling pathway was affected only in the colon cancer cells. Notably, WNT target genes were similarly downregulated after treatment of HCT116 with C646 as with ICG-001.

**Conclusion:**

Our results suggest that treatment with a general HAT inhibitor causes similar effects on the transcriptome as does treatment with a CBP-specific inhibitor and that epigenetic inhibition affects the WNT pathway in HCT116 cells and the cholesterol biosynthesis pathway in PANC1 cells.

**Electronic supplementary material:**

The online version of this article (doi:10.1186/1756-8935-8-9) contains supplementary material, which is available to authorized users.

## Background

Due to the hyper-activation of WNT signaling in a variety of cancer types [1, 2], there has been a strong drive to develop antagonists of WNT signaling for cancer treatment. Standard inhibitors of the WNT signaling pathway include biologic inhibitors, such as small interfering RNAs, antibodies, and recombinant proteins, and chemical inhibitors, such as NSAIDs, vitamins, and polyphenols, that have fairly general (or unknown) targets [1, 3, 4]. However, a new category of drugs to target the WNT pathway is being developed that holds high promise as chemotherapeutics. These drugs, called epigenetic inhibitors, function to modify chromatin structure. Chromatin is composed of nucleosomes, which are comprised of 146 bp of DNA wrapped around eight core histone proteins (two copies each of H2A, H2B, H3, and H4). The N terminal tails of the core histones that constitute the nucleosome are subject to various different types of modifications that can influence chromatin structure and either enhance or inhibit the ability of transcription factors to bind to and regulate their target genes. The pattern of histone modifications throughout the genome, in combination with the pattern of DNA methylation, is called the epigenome. Recent studies have revealed that different histone modifications are associated with active vs. silenced chromatin, that different cell types show different epigenomic patterns of silenced vs. active chromatin, and that changes in chromatin structure can have a dramatic effect on cell proliferation, differentiation, and survival. One widely studied histone modification is acetylation; histone acetylation is a critical regulatory mechanism of gene expression and plays an important role in gene expression. In fact, acetylation of histone H3 on lysine 27 is the epigenetic modification that most precisely identifies distal regulatory regions that serve as active enhancers [5]. Because cancer genomes show changes in histone acetylation patterns, there is great interest in the use of acetylation inhibitors that inhibit signaling pathways linked to human cancers for epigenetic therapy [6].

**Figure 1:**
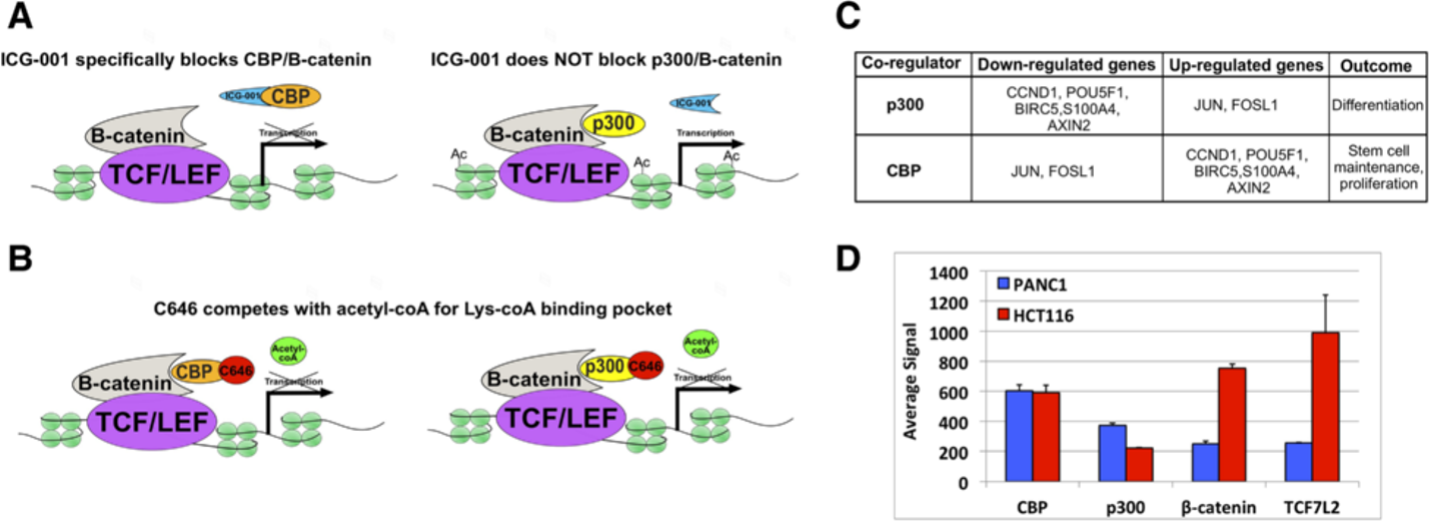
**Targeting the WNT pathway using epigenetic inhibitors.** WNT signaling culminates when, upon recruitment of β-catenin/CBP or β-catenin/p300 complexes to the DNA via a TCF/LEF family member, CBP and p300 activate transcription by acetylating histone H3. **(A)** Treatment with ICG-001 disrupts the interaction of CBP with β-catenin, blocking CBP-driven, but not p300-driven transcription. **(B)** In contrast to the effects of ICG-001, C646 competes with acetyl-coA for the Lys-coA binding pocket of both CBP and p300, preventing HAT activity of both complexes. **(C)** Examples of predicted gene expression differences mediated by β-catenin/CBP vs. β-catenin/p300 complexes [15]. **(D)** RNA levels in HCT116 and PANC1 cells of the various components of the WNT signaling model.

Drugs that inhibit acetylation are particularly relevant for inhibition of the WNT pathway. The canonical WNT signaling pathway initiates when WNT ligands bind to receptors, resulting in the nuclear localization of the co-activator β-catenin (CTNNB1), which leads to an association of β-catenin with a member of the TCF/LEF transcription factor family at regulatory regions of WNT-responsive genes [7, 8]. The TCF/β-catenin complex can interact with co-activators such as CBP (CREBBP) and p300 (EP300) which function in part through the acetylation of histone H3 on lysine 27 [5]. Thus, it has been proposed that the initiation of the WNT signaling pathway ultimately ends with histone acetylation and a relaxing of the chromatin structure, a process necessary for gene activation. The small molecule inhibitor ICG-001 binds to CBP and competitively inhibits the interaction of CBP with β-catenin [9, 10], with the expected result of loss of active histone at promoters and enhancers regulated by TCF/β-catenin/CBP complexes (Figure 1A). Importantly, ICG-001 does not bind to the highly related histone acetyltransferase (HAT) p300 and should not affect the activity of promoters or enhancers bound by TCF/β-catenin/p300 complexes. Thus, ICG-001 is thought to specifically decrease the expression of only the subset of WNT target genes regulated by β-catenin/CBP interactions. These proposed effects of ICG-001 are in contrast to those of C646 an inhibitor that competes with acetyl-coA for the Lys-coA binding pocket of p300 (Figure 1B). C646 is very selective for p300 versus six other unrelated histone acetyltransferases [11]. Although no direct comparisons have been performed, due to the mode of action of C646 and because the HAT domains of p300 and CBP have greater than 90% similarity, it has been proposed that C646 is a general inhibitor for both CBP and p300 [11]. Of importance for the role of ICG-001 as a chemotherapeutic drug, studies suggest that CBP-driven transcription helps to maintain pluripotency whereas p300-driven transcription pushes cells toward a differentiated state [3, 12–15]; examples of genes thought to be regulated by CBP vs. p300 are shown in Figure 1C. However, the hypothesis that ICG-001 specifically downregulates only the subset of WNT target genes involved in proliferation (such as *BIRC5* and *CCND1*) has not been tested on a genome-wide scale. Because a derivative of ICG-001 called PRI-724 is now in clinical trials (NCT01302405 and NCT01606579), it is critical to have a thorough understanding of the specificity and effectiveness of this drug. Therefore, we have compared the genome-wide effects on the transcriptome of ICG-001 versus C646 in two cancer cell lines that constitutively express the components of the transcription complex that mediates WNT signaling (Figure 1D).

## Results

### ICG-001 and C646 have similar effects on the transcriptome of HCT116 colon cancer cells

Constitutive activation of WNT target genes via a TCF/β-catenin/CBP complex is thought to be a major driver of colorectal cancer. Therefore, it has been proposed that treatment of colon cancer cells with ICG-001 should specifically inhibit the WNT pathway (by preventing recruitment of the co-activator CBP to TCF/β-catenin target genes) and reduce the tumorigenicity of the cells. In support of this hypothesis, Emami *et al.*[10] have shown that ICG-001 reduces growth of colon carcinoma cells in culture and reduces the formation of colon and small intestinal polyps in a mouse model system. As noted above, CBP is highly related to another HAT called p300 and many studies have shown similar functions for p300 and CBP [16]. In fact, a ChIP-seq analysis of p300 and CBP in T98G glioblastoma cells immediately after release from serum starvation arrest showed that almost all of the CBP genomic binding sites were also bound by p300 [17]. However under the tested conditions, a small set of genomic sites were preferentially bound by either CBP or p300, suggesting that there might be some specificity in their action. It is also possible that cell type plays a critical role in specifying CBP vs. p300 contributions to regulating the transcriptome. For example, approximately 50% of Rubinstein-Taybi syndrome patients have mutations in CBP but only 3% of patients have mutations in p300 [18]. Of course, functional specificity can also occur post-DNA binding because the two HATs only share extensive, but not complete, homology. If, for example, CBP and p300 recruit different interaction partners they could have opposite effects on transcription at a given promoter. In support of this hypothesis, Ma *et al.* have shown that both CBP and p300 can bind to the *BIRC5* promoter but they have opposite effects on transcription [19].

**Figure 2:**
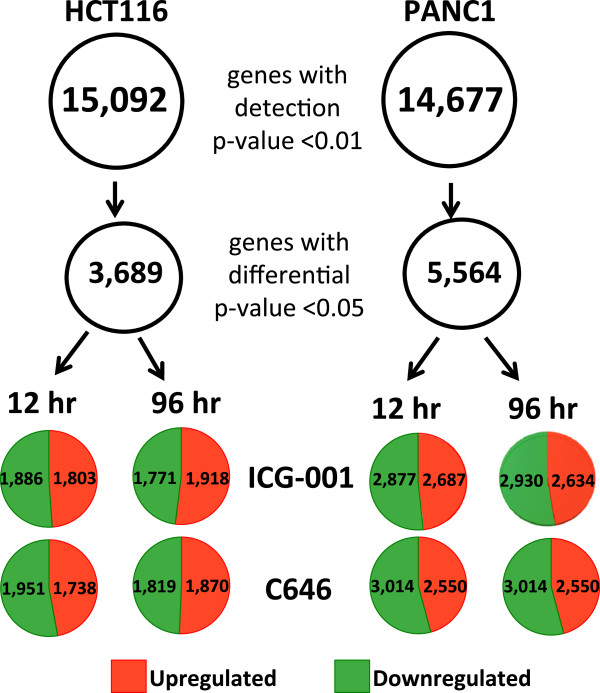
**The effects of epigenetic inhibitors on the transcriptome of HCT116 and PANC1 cells.** HCT116 colon cancer cells and PANC1 pancreatic adenocarcinoma cells were treated in duplicate with DMSO or 10 uM ICG-001 or C646 for 12 or 96 h (12 samples per cell line). Cells were harvested and RNA was analyzed using Illumina HumanHT-12 v4 expression arrays. Any gene having a detection *P* value <0.01 in any of the samples was selected for differential gene analysis; genes having a differential *P* value <0.05 were further analyzed. The number of upregulated (red) and downregulated (green) genes under each condition for each cell line is shown.

**Figure 3:**
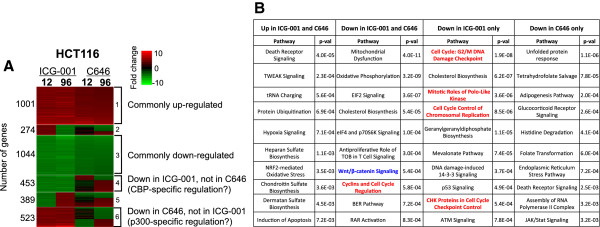
**Effects of epigenetic inhibitors on gene expression in HCT116 cells. (A)** Genes differentially expressed after treatment of HCT116 cells with ICG-001 or C646 (see Figure 2) were analyzed using Euclidean distance and K-means clustering of expression fold change. **(B)** Gene ontology analyses are shown for the genes commonly up- and downregulated by both drugs and for the genes that are downregulated only by one of the drugs in HCT116 cells. Terms related to the cell cycle are shown in red and terms related to WNT signaling are shown in blue. The numbers 1 to 6 in the brackets in panel A refer to different clusters that were used in the gene ontology analyses shown in panel B (see Additional file 3).

To determine if the effects on the transcriptome after specifically inhibiting CBP are different than the effects after inhibiting both CBP and p300, we treated HCT116 colon cancer cells with 0.05% DMSO, 10 uM ICG-001, or 10 uM C646 for 12 and 96 h. Samples were prepared in replicate and Illumina HumanHT-12 v4 expression arrays were used to detect changes in gene expression (Figure 2 and Additional file 1). Genes having a detection *P* value less than 0.01 in any of the control or treated cell populations were selected for further analysis; this constituted a total of 15,092 genes from HCT116 cells, of which 3,689 showed differential expression in drug-treated cells (differential expression *P* value less than 0.05). After selecting the significant differentially expressed genes, the expression fold change was calculated for each gene and Euclidean distance was used for K-means clustering of expression fold change (Figure 3). We found that, contrary to our initial expectations, a very similar response was observed for both drugs (Additional file 2). Genes that were downregulated by both drugs were involved in the cell cycle and WNT signaling (Figure 3 and Additional file 3). However, some genes did show drug-specific changes in HCT116 cells. According to the mechanism of action of each drug, genes with decreased levels of expression only after treatment with ICG-001 should be regulated by CBP but not by p300, whereas genes with decreased levels of expression only after treatment with C646 but not with ICG-001 should be regulated by p300 but not by CBP. A gene ontology analysis of the approximately 400 genes affected only by ICG-001 revealed a strong enrichment for genes controlling the cell cycle whereas the approximately 500 genes only affected by C646 were not related to cell proliferation. Thus, in HCT116 cells, both drugs have a broad effect on gene regulation that includes downregulation of genes involved in proliferation control. However, treatment of colorectal cancer cells with ICG-001 alters the expression of a greater number of cell cycle-regulated genes than does treatment with C646.

### ICG-001 and C646 have similar effects on the transcriptome of PANC1 cells

**Figure 4:**
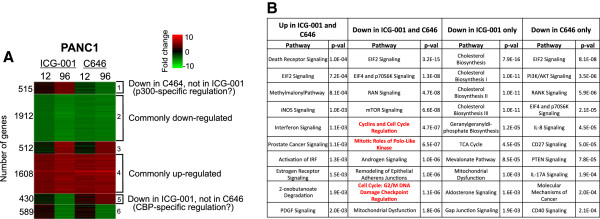
**Effects of epigenetic inhibitors on gene expression in PANC1 cells. (A)** Genes differentially expressed after treatment of PANC1 cells with ICG-001 or C646 (see Figure 2) were analyzed using Euclidean distance and K-means clustering of expression fold change. **(B)** Gene ontology analyses are shown for the genes commonly up- and downregulated by both drugs and for the genes that are downregulated only by one of the drugs in PANC1 cells. Terms related to the cell cycle are shown in red. The numbers 1 to 6 in the brackets in panel A refer to different clusters that were used in the gene ontology analyses shown in Panel B (see Additional file 4).

As noted above, the WNT/TCF/β-catenin/CBP pathway has been proposed to be a major positive regulator of proliferation of colon cancer cells. Perhaps β-catenin/CBP complexes play a prominent role in WNT-mediated gene expression in HCT116 cells (with little contribution by β-catenin/p300 complexes), explaining why the effects of ICG-001 were so widespread and why treatment with the two drugs elicited similar responses. To determine if ICG-001 has a similar widespread effect on other cancer cells, we also examined pancreatic cancer cells. Pancreatic ductal adenocarcinoma, the most common form of pancreatic cancer, displays activation of the WNT/β-catenin pathway [20–25] and is therefore predicted to respond to treatment with ICG-001. We treated PANC1 cells with ICG-001 or C646 and analyzed gene expression (Additional files 1 and 2). Again, we found that ICG-001 and C646 have similar effects on PANC1 cells (Figure 4), with genes involved in cell cycle regulation being downregulated by both drugs (see also Additional file 4). However, in this case, cell proliferation-related genes were not enriched categories in gene sets downregulated specifically by either ICG-001 or C646. Interestingly, in PANC1 cells, the cholesterol biosynthesis pathway was highly enriched for genes specifically downregulated by ICG-001, suggesting that perhaps genes involved in cholesterol biosynthesis are specific CBP, but not p300, target genes. In contrast, p300-specific genes (identified as those responsive only to C646) appear to be involved in various types of signaling pathways, including PI3K/AKT signaling which is linked to cell survival.

**Figure 5:**
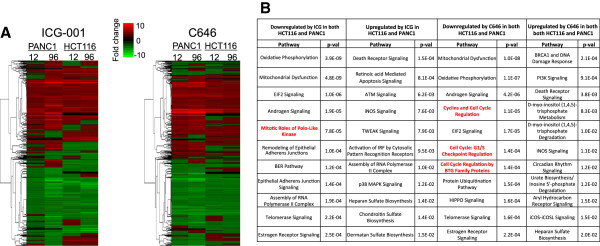
**ICG-001 and C646 affect many of the same genes in HCT116 and PANC1 cells. (A)** Genes that were significantly detected in both HCT116 or in PANC1 cells (*P* value <0.01) were analyzed for expression differences caused by drug treatment. All genes having a differential *P* value <0.05 and a fold change greater than 1.2 were analyzed using Euclidean distance and hierarchical clustering. **(B)** Gene ontology analyses are shown for the genes commonly up- or downregulated in HCT116 and PANC1 cells by the drugs. Terms related to the cell cycle are shown in red. See Additional file 5 for a complete gene ontology analysis of the up- and downregulated genes.

To determine if gene responses to the drugs were cell type-specific, we compared the genes whose expression was altered by ICG-001 or C646 in both HCT116 and PANC1 cells (a total of 6,732 genes). Genes that were significantly detected in HCT116 or in PANC1 cells (*P* value <0.01) and which had a differential *P* value <0.05 and a fold change greater than 1.2 (5,182 genes) were compared using hierarchical clustering with Euclidean distance and average linkage measures (Figure 5). We found that although some genes were altered in a cell type-specific manner, most genes were similarly affected in both cell types; see Additional file 5 for a list of the cell type-specific and cell-type common affected genes. A gene ontology analysis revealed that the top two categories of genes downregulated by ICG-001 or C646 in both HCT116 and PANC1 cells were oxidative phosphorylation and mitochondrial dysfunction. Genes that were commonly upregulated by the drugs in both cell types are involved in pathways such as death receptor signaling and INOS signaling.

### Effectiveness of the epigenetic inhibitors in reverting a tumor cell phenotype

**Figure 6:**
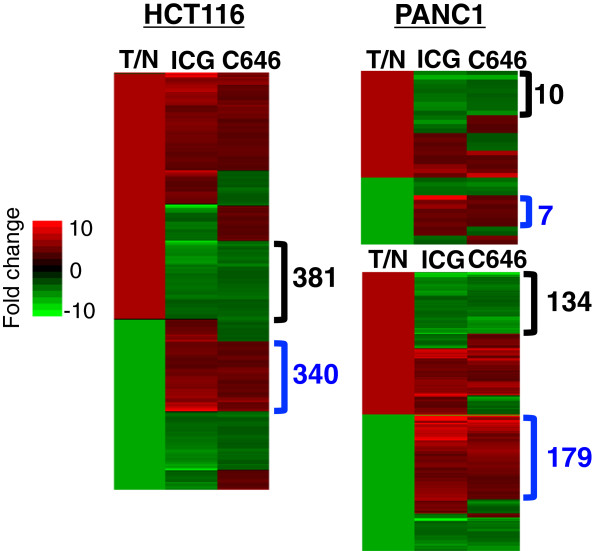
**Epigenetic inhibitors can partially restore a normal expression pattern to tumor cells.** Genes that showed tumor-specific changes in expression in TCGA colon RNA-seq samples **(left)**, TCGA pancreatic RNA-seq samples **(right, top)**, plus differentially expressed genes identified by comparison of normal to tumor pancreatic cell lines **(right bottom)** were analyzed for responses to drug treatments; see Additional file 6. In the T/N columns, green indicates that the gene was downregulated in the tumor cells whereas red indicates the gene was upregulated in the tumor cells. The blue brackets indicate genes that were downregulated in the tumor cells and upregulated by the drugs (resulting in an expression level closer to that in normal cells) whereas the black brackets represent the genes that were upregulated in the tumor cells and downregulated by the drugs (resulting in an expression level closer to that in normal cells). The color scale indicates the fold change of gene expression in HCT116 or PANC1 cells after treatment with ICG-001 (ICG) or C646.

The ultimate goal of epigenetic therapy is to revert the transcriptome from a tumor-specific pattern of gene expression back to the expression patterns seen in normal cells. To determine the extent to which the epigenetic inhibitors ICG-001 and C646 are effective in this goal, we obtained RNA-seq expression data for 41 normal and 274 tumor colon cells from the TCGA Consortium. Using this data, we identified 16,416 genes that were expressed in either normal or colon samples, using log2(RSEM + 1) >2. Of these, 11,824 genes were differentially expressed (adjusted differential *P* value <0.001) in the tumor samples as compared to the normal tissues. To determine if the drugs were effective in reverting the expression of these genes back to normal levels, we compared the set of genes deregulated in the tumors with the set of genes responsive to the drug treatments, identifying a set of 2,028 common genes. If the drugs are having an anti-tumor effect, then genes that are upregulated in tumors should be downregulated by the drugs and genes that are downregulated in tumors should be upregulated by the drug. Using a log2(RSEM + 1) cutoff of 2, we identified 2,029 genes that showed expression changes (adjusted *P* value <0.05) in colon tumor cells, as compared to the normal tissues. An analysis of these expression patterns (Figure 6 and Additional file 6) shows that many genes had expression changes in the correct direction as a result of treatment with at least one drug (that is, a gene that is upregulated in tumors was downregulated by a drug or a gene that is downregulated in tumors was upregulated by a drug). Analysis of four normal and 125 pancreatic tumor samples revealed a much smaller set of genes showing expression changes in tumors. Using a log2(RSEM + 1) cutoff of 2, we identified only 167 genes that showed expression changes (adjusted *P* value <0.05) in pancreatic tumor cells, as compared to the normal tissues. It is unclear as to whether the small number of differentially expressed genes in the pancreatic tumors as compared to the colon tumors is due to real differences in cancer phenotypes, to the small number of normal pancreatic samples, or other possibilities such as tumor heterogeneity. To increase the number of analyzed genes, we also obtained a list of 596 genes that are differentially expressed in normal hTERT-HPNE pancreatic cells as compared to PANC1 cells [26]. We examined the responses of the 167 genes that are differentially regulated in normal pancreatic tissue vs. tumors and the 596 genes that are differentially regulated in normal HPNE cells grown in culture vs. PANC1 cells to drug treatment. We found that many of the genes whose expression is deregulated in pancreatic tumors or PANC1 cells showed appropriate responses to at least one drug (that is, genes upregulated in tumors or PANC1 were downregulated by the drugs and genes downregulated in tumors or PANC1 were upregulated by the drugs) (Figure 6 and Additional file 6). Thus, treatment with the epigenetic inhibitors is effective in reverting some of the tumor-specific transcriptome to a normal pattern.

### Direct targeting of a component of the transcription complex that mediates WNT signaling

**Figure 7:**
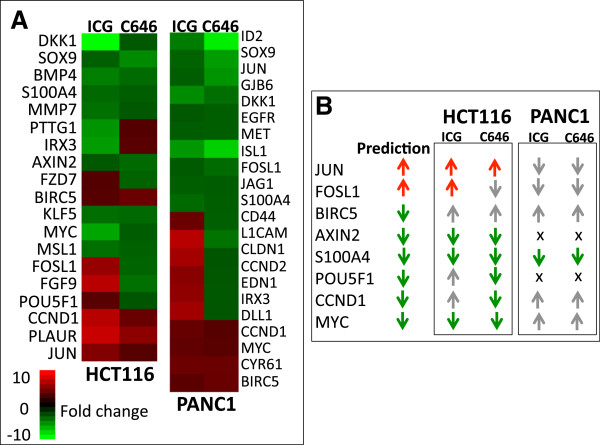
**Effects of drug treatments on WNT pathway genes. (A)** Shown are the expression changes in previously identified WNT pathway genes (http://www.stanford.edu/group/nusselab/cgi-bin/wnt/) that have a detection *P* value <0.01 and a differential *P* value <0.05 after 96 h of treatment of HCT116 or PANC1 cells with either ICG-001 (ICG) or C646; see Additional file 7. **(B)** Shown are the predicted results (based on the model shown in Figure 1) and the actual responses to the drugs after treatment of HCT116 or PANC1 cells for a set of WNT target genes. In the prediction column, a red arrow indicates that the gene should have been upregulated by ICG-001 and the green arrow indicates that the gene should have been downregulated by ICG-001, according to the model. For each cell type, the actual response is shown for both drugs: a red arrow indicates that expression was increased as predicted by the model, a green arrow indicates expression was decreased as predicted by the model, a gray arrow indicates that the expression pattern upon treatment did not correspond to the prediction, and an x indicates that the gene was not expressed in that cell line.

As described above, ICG-001 was developed to be a specific inhibitor of the WNT pathway. We therefore directly analyzed the WNT pathway using a list of genes previously implicated as components of this pathway (http://www.stanford.edu/group/nusselab/cgi-bin/wnt/). We found that a subset of these proposed WNT target genes were expressed in HCT116 and/or PANC1 cells and were significantly affected by treatment with ICG-001 or C646 (Figure 7 and Additional file 7). The overall trend of the effects of ICG-001 and C646 on WNT targets was similar in a given cell line. However, the WNT pathway-related genes responded quite differently to the epigenetic inhibitors in the different cell lines. In general, the response of the genes listed in Figure 1C was more similar to what was predicted when HCT116 cells were treated with the epigenetic inhibitors than when PANC1 cells were treated with the drugs. For example, expression of the transcription factor *JUN* (which is involved in specifying differentiated phenotypes) is increased by both drugs in HCT116 but is decreased by both drugs in PANC1. Conversely, the expression of *MYC*, a transcription factor involved in cell proliferation, is reduced by both drugs in HCT116 but is increased by both drugs in PANC1 cells.

**Figure 8:**
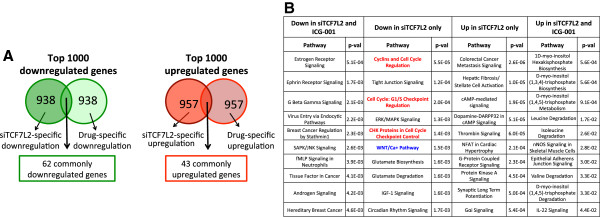
**In PANC1 cells, treatment with ICG-001 does not affect the same genes as does reduction in levels of TCF7L2. (A)** PANC1 cells were treated with siRNAs to *TCF7L2* and RNA-seq was performed; see Additional file 8 for a list of genes affected by knockdown of *TCF7L2*. The top 1,000 differentially expressed genes after knockdown of *TCF7L2* were compared to the top 1,000 genes identified to be responsive to ICG-001 in PANC1 cells. **(B)** Gene ontology analyses are shown for the genes commonly up- and downregulated by knockdown of *TCF7L2* and treatment with ICG-001 and genes that are only affected by knockdown of *TCF7L2*. Terms related to the cell cycle are shown in red and terms related to the WNT pathway are shown in blue. See Additional file 9 for a complete gene ontology analysis of the different gene sets.

The gene ontology results suggest that ICG-001 and C646 affect the WNT pathway in HCT116 cells but not in PANC1 cells. Of course, it is also possible that different downstream target genes mediate the WNT pathway in pancreatic cancer cells as compared to colon cancer cells. The HATs CBP and p300 are brought to genomic regulatory elements by the DNA binding protein TCF7L2 via interaction with the bridging protein β-catenin. If ICG-001 and C646, which block the recruitment or function of the HAT activity of the co-activators CBP and p300, are specific inhibitors of the WNT signaling pathway in PANC1 cells, then targeting TCF7L2 should result in similar effects on the transcriptome as does drug treatment. In contrast, if the epigenetic inhibitors are in fact targeting a different pathway in PANC1 cells, then genes affected by reduction of TCF7L2 should be different than the set of genes affected by the drugs. To identify genes affected by direct targeting of a component of the transcriptional complex implicated in WNT regulation, we used siRNAs to knockdown *TCF7L2* in PANC1 cells. Cells were treated with control siRNAs or siRNAs specific for *TCF7L2* and RNA was analyzed by RNA-seq; see Additional file 8 for a list of genes affected by knockdown of *TCF7L2*. We analyzed the top 1,000 genes that were affected by knockdown of *TCF7L2* and the top 1,000 genes affected by treatment with ICG-001 (Figure 8). Interestingly, there were very few genes affected by reduction of *TCF7L2* that were also affected by ICG-001. Specifically, the WNT pathway was identified in the set of genes affected upon reduction of *TCF7L2* (see Additional file 9 for a complete gene ontology analysis) but not by treatment with ICG-001 (Figure 4). These results suggest that in PANC1 cells co-activators other than CBP cooperate with TCF7L2 to regulate gene expression and support the hypothesis that the anti-proliferative effects of ICG-001 in PANC1 cells are not due to inhibition of the WNT pathway.

## Discussion

Recent studies have shown large changes in the epigenomic patterns in normal vs. cancer cells, suggesting that epigenetic therapy may be commonly applicable to treatments of various cancers. Drugs that target epigenetic regulators are being developed [27–29], some of which are moving into clinical trials. However, the specificity of action of many of these drugs has not yet been thoroughly examined. In particular, genome-wide analyses of their effects have not been determined. In our study, we compare the effects of treatment with C646, which is thought to compete with acetyl-coA for the Lys-coA binding pocket of both p300 and CBP [11] to the effects of ICG-001, which specifically binds to CBP and prevents its interaction with the co-activator β-catenin. Theoretically, ICG-001 is expected to be of higher specificity than C646 because it should only affect β-catenin/CBP-driven transcription whereas C646 should affect all genes regulated by either CBP or p300, regardless of whether β-catenin is involved. However, it is possible that ICG-001 has broader effects than anticipated if the drug affects the ability of CBP to interact with other as-of-yet unknown co-activators. In addition, we note that CBP and p300 can acetylate non-histone proteins [30]; thus, both compounds could also have effects on non-chromatin bound proteins. Although we initially expected cells to respond differently to C646 and ICG-001, our results suggest that generally these two drugs have similar effects on the transcriptome of tumor cells. However, we did identify some cell-specific and drug-specific responses after epigenetic inhibition.

We observed dramatic effects on the transcriptome upon treatment of HCT116 colon cancer cells with either ICG-001 or C646, with thousands of genes showing differential expression. Interestingly, the responses to the two drugs were quite similar overall, with both drugs causing a reduction in certain genes involved in the WNT pathway. Because ICG-001 affects only CBP-driven transcription and not p300-driven transcription, these results suggest that perhaps the majority of the WNT-related active regulatory elements in HCT116 cells are bound by β-catenin/CBP complexes. We did identify a set of approximately 500 genes whose expression was downregulated by ICG-001 and not by C646 (these are potential CBP-specific target genes) and a set of approximately 500 genes whose expression was downregulated by C646 but not by ICG-001 (these are potential p300-specific target genes). These results are similar to a previous study of CBP and p300 in T98G glioblastoma cells that found that the two factors bound mainly to the same sites but that some specific binding sites could be identified [17]. Interestingly, the genes specifically responsive to ICG-001 but not to C646 in HCT116 cells showed enrichment for cell proliferation-related gene ontology categories. Taken together, these results suggest that thousands of genes are regulated both by p300 and CBP (many of which are involved in cell proliferation) and that CBP-specific genes may also include additional genes that regulate cell proliferation whereas p300-specific genes are involved in other processes. In general, our results in HCT116 cells support the current model implicating WNT-mediated cell signaling as a critical regulator of cancer cell proliferation.

Although the WNT pathway has been implicated in the development of pancreatic cancer, the studies are not as extensive as those related to WNT’s role in colon cancer [20–25]. We show that, in general, the effects of ICG-001 and C646 on the transcriptome of PANC1 cells are similar to those observed upon treatment of HCT116 cells. For example, a set of genes involved in cell proliferation show reduced expression upon treatment of PANC1 with either ICG-001 or C646. However, we did observe several differences in the response of PANC1 cells to the epigenetic inhibitors, as compared to HCT116 cells. First, we found that many of the enriched gene categories that responded specifically to ICG-001 treatment of PANC1 cells are involved in cholesterol biosynthesis. Interestingly, many cancers have a high dependency on accelerated biogenesis and uptake of lipids and cholesterol and inhibition of these pathways has been proposed to be a therapeutic opportunity for metabolic targeting of cancer growth [31, 32]. Cholesterol homeostasis in mammalian cells is maintained in part by a basic-helix-loop-helix family of transcription factors called the sterol regulatory element binding proteins (SREBPs) [33, 34]. The SREBP family members activate a number of target genes involved in cholesterol and fatty acid metabolism through binding to sterol regulatory elements in the promoters of target genes. In fact, SREBP transcription factors have been suggested to be novel therapeutic targets [35]. Interestingly, SREBP proteins require interaction with CBP to mediate transcriptional activation [36]. Thus, the treatment of PANC1 cells with ICG-001 likely disrupts a functional interaction between CBP and a SREBP family member, causing downregulation of genes involved in the cholesterol biosynthetic pathway (Additional file 10). Second, in PANC1 cells the WNT pathway was not enriched in downregulated genes after treatment with either drug and several critical WNT target genes showed unexpected transcriptional responses. Notably, expression of *JUN* (which promotes differentiation) was predicted to be increased upon treatment but in PANC1 cells *JUN* expression was decreased (*JUN* did show the expected response in HCT116 cells). Similarly, expression of *MYC* (which promotes proliferation) was predicted to be decreased upon treatment but in PANC1 cells *MYC* expression was increased (*MYC* did show the expected response in HCT116 cells). The transcriptional response of the *MYC* gene was particularly surprising because it is considered to be a critical mediator of WNT signaling. Upregulation of *MYC* in PANC1 suggests that the drugs do not inhibit the WNT pathway in these cells. This hypothesis is supported by our finding that in PANC1 cells knockdown of TCF7L2, the transcription factor that brings β-catenin and CBP to regulatory elements to regulate WNT-responsive genes, does not affect expression of the same genes as are affected by treatment with ICG-001. While our work was in progress, another group reported treatment of pancreatic cancer cells with ICG-001 [37]. They showed that treatment of PANC1 cells with 10 uM ICG-001 was effective at reducing cell proliferation in culture and reducing colony formation in soft agar. Although global effects on the PANC1 transcriptome were not examined in that study, the noted effects on proliferation are consistent with our finding that cell cycle-related genes are downregulated in response to ICG-001 and C646. That study did, however, perform microarray expression analysis after treatment of a different pancreatic cancer cell line (AsPC-1) with ICG-001 and found that 569 transcripts were upregulated and 150 transcripts were downregulated. Because only 117 of the 719 drug-responsive genes were altered in β-catenin knockdown cells, they concluded that ICG-001 had a broader effect than simply as a disrupter of WNT/β-catenin signaling in AsPC-1 cells.

As noted above, epigenetic inhibitors are considered promising new drugs for cancer treatment. One current clinical trial employs PRI-724, a derivative of ICG-001, in combination with gemcitabine in patients with advanced or metastatic pancreatic adenocarcinoma (NCT01764477). Gemcitabine is considered a first-line treatment for pancreatic adenocarcinoma but has poor overall efficacy because pancreatic cancer cells develop resistance to the drug [38]. While investigating the pathways that lead to drug resistance, the transcriptional regulator NUPR1 (also known as anti-apoptotic protein p8 or Candidate of Metastasis-1) was identified as being involved in the acquisition of gemcitabine resistance by pancreatic cancer cells [39]. NUPR1 normally functions as a stress response gene in the pancreas, but it has been shown to contribute to metastasis, anti-apoptotic activity and pancreatic cancer development [40, 41]. Interestingly, our genome-wide analyses identified *NUPR1* as one of the top upregulated genes after treatment of PANC1 cells with ICG-001. The upregulation of *NUPR1* by ICG-001 may explain why ICG-001 plus gemcitabine did not increase overall lifespan in an *in vivo* pancreatic cancer cell xenograft model [37]. Although the mechanism by which NUPR1 promotes oncogenesis and/or drug resistance in pancreatic cells is not yet known, NUPR1 has been shown to form a complex with p300 and TP53 to upregulate and promote cytoplasmic translocation of CDKN1A (p21) in breast cancer cells [42]. Although nuclear p21 is a negative regulator of cell cycle progression, studies have associated cytoplasmic p21 with drug resistance and oncogenic activity in breast and testicular cancer [43–45]. Vincent *et al.*[44] showed that treatment of NUPR1-expressing cells with PI3K-AKT inhibitors could reverse cytoplasmic p21 localization and re-sensitize cells to doxorubicin. Importantly, studies have also shown that inhibition of the PI3K-AKT pathway in pancreatic cancer helps re-sensitize cells to gemcitabine [46, 47]. Thus, adding a PI3K-AKT inhibitor to the combined usage of ICG-001 plus gemcitabine may be the most effective treatment combination. However, it should also be noted that C646 caused only a modest increase in *NUPR1* in PANC1 cells and that C646, but not ICG-001, specifically inhibited the PI3K-AKT pathway (see Figure 4). Taken together, these results suggest that perhaps C646 plus gemcitabine would be more effective than ICG-001 plus gemcitabine in the treatment of pancreatic cancer.

## Conclusions

We have compared the genome-wide effects on the transcriptome of ICG-001 (a specific CBP inhibitor) versus C646 (a compound that competes with acetyl-coA for the Lys-coA binding pocket of both CBP and p300). We found that ICG-001 has a similar broad specificity as C646 in HCT116 colon cancer, with both drugs decreasing the expression of cell cycle-related and WNT pathway genes. In contrast, ICG-001 and C646 affect cell cycle-related genes but do not result in appropriate responses of critical WNT target genes in PANC1 cancer cells. The effects of ICG-001 on PANC1 cells and comparison to gene expression patterns in TCF7L2 knockdown cells suggests that ICG-001 inhibits proliferation of pancreatic cancer cells via a mechanism different than the WNT pathway. Gene ontology analyses point toward disruption of SREBP-CBP functional interactions as a possible cause of the anti-proliferative function of ICG-001 in pancreatic cancer cells. Importantly, both epigenetic inhibitors are effective at reversing some tumor-specific changes in gene expression that are observed in colon or pancreatic tumor cells.

## Methods

### Cell growth conditions

The human cell lines HCT116 (ATCC #CCL-247) and PANC1 (ATCC #CRL-1469) were obtained from the American Type Culture Collection. HCT116 and PANC1 cells were grown in Dulbecco’s modified Eagle’s medium supplemented with 10% fetal bovine serum and 1% penicillin/streptomycin. Michael Kahn (University of Southern California) provided ICG-001 and C646 was obtained from VWR (catalog # 102516–240). Cells were treated with 10 uM ICG-001, 10 uM C646, or 0.05% DMSO and collected after 12 or 96 h. Cells for the 12-h treatments were grown to 70% confluency before addition of the drugs or DMSO. Cells for the 96-h treatments were grown at 40% to 50% confluency before addition of the drugs or DMSO and were passaged before they could reach 90% confluency. New media and drugs were added every 24 h. After treatment, gene expression was analyzed using Illumina BeadChips.

### Microarray RNA expression

Total RNA was collected using Trizol according to the manufacturer’s instructions (Life Technologies). To confirm RNA samples were not degraded, RNA quality was checked with the Experion StdSens kit (Bio-Rad) prior to amplification and labeling. The Illumina TotalPrep RNA Amplification Kit (Life Technologies catalog # AMIL1791) was used according to the manufacturer’s instructions to amplify and label RNA samples for Illumina array hybridization. Labeled RNAs were analyzed with Illumina HT-12 v4 Expression BeadChips (Catalog #: BD-103-0204) with the Direct Hybridization Assay and then scanned on an Illumina HiScan (catalog # BD-103-0604). The data were analyzed and exported from Illumina’s GenomeStudio software using quantile normalization without background subtraction. Each drug/DMSO treatment and time point was performed using two independent biological replicates. The correlation between replicates was calculated to ensure that the data were reproducible, replicate samples were averaged together and genes with a detection *P* value <0.01 were considered for further analysis. Differential expression analysis was performed using Illumina’s custom differential expression error model, which assumes a normal distribution of the target signal intensity and takes into account biological variation, non-specific biological variation, and technical error. For more detail on Illumina’s custom error model, see GenomeStudio Gene Expression Module v1.0 User Guide (pages 103 and 104). Genes with a differential expression *P* value <0.05 were considered to be significantly differentially expressed.

### *TCF7L2* knockdown

*TCF7L2* knockdown was performed in triplicate by siRNA transfection. Transfections were performed using Lipofectamine RNAiMax (Life Technologies) according to manufacturer’s instructions. A final concentration of 40nM siRNAs targeting either *TCF7L2* (catalog # 4392420, Life Technologies) or a non-specific negative control siRNA (catalog # AM4611, Life Technologies) were used using reduced serum OptiMEM media (Life Technologies). Media was changed 12 h post transfection and total RNA was collected 48 h post transfection using Trizol according to manufacturer’s instructions (Life Technologies). Knockdown efficiency was detected using RT-qPCR and then samples were analyzed by RNA-seq.

### RNA-Seq

Total RNA was used for polyA+ RNA selection using oligo-dT beads and subjected to library construction by True-Seq library preparation kits (Illumina), followed by Illumina HiSeq2000 sequencing. The RNA-seq reads were aligned to the human genome hg19 using Bowtie2 with ultrasensitive parameters. The RNA-seq reads were counted over gene exons using HTSeq [48]. EdgeR was used for statistical analyses of siControl and siTCF7L2 samples, and a fold change of 2 was used to call the differentially expressed genes [49].

### Ingenuity pathway analysis

Gene network diagrams in Additional file 10 were created through use of IPA. The expression data were analyzed through the use of QIAGEN’s Ingenuity® Pathway Analysis (IPA®, QIAGEN Redwood City, http://www.qiagen.com/ingenuity). For each subset of genes a core analysis was run with parameters set to consider only direct relationships and relationships between molecules that have been experimentally observed. The reference gene set used for *P* value calculations was the Ingenuity Knowledge Base (genes only).

### Data access

Expression array analyses for control and treated cells and RNA-seq datasets for *TCF7L2* knockdown experiments have been deposited in GEO (GSE64039 and GSE63776). The TCGA RNA-seq can be downloaded at https://tcga-data.nci.nih.gov/tcga/tcgaDownload.jsp.

## Electronic supplementary material

Additional file 1: **(A) Gene expression analysis after drug treatment of HCT116 cells.** (B) Gene expression analysis after drug treatment of PANC1 cells. (XLSX 19 MB)

Additional file 2: **Genes with expression changes after drug treatment.** (XLSX 656 KB)

Additional file 3: **Gene ontology analysis of HCT116-treated cells.** (XLSX 69 KB)

Additional file 4: **Gene ontology analysis of PANC1-treated cells.** (XLSX 82 KB)

Additional file 5: **Cell type-specific and common gene expression responses.** (XLSX 578 KB)

Additional file 6: **Drug responses of genes altered in tumors.** (XLSX 348 KB)

Additional file 7: **WNT target gene response to ICG-001.** (XLSX 44 KB)

Additional file 8: **Gene expression after TCF7L2 knockdown in PANC1.** (XLSX 550 KB)

Additional file 9: **Gene ontology analysis of siRNA vs. drugs in PANC1.** (XLSX 456 KB)

Additional file 10: **Cholesterol biosynthesis pathway.** (PDF 1 MB)

## Abbreviations

HAT: Histone acetyltransferase
hTERT-HPNE: Normal human pancreatic ductal cells immortalized with TERT
SREBP: Sterol regulatory element binding protein.
