# Supplementary material for: Altering cancer transcriptomes using epigenomic inhibitors
Source: Epigenetics Chromatin. 2015 Feb 24;8:9. doi: 10.1186/1756-8935-8-9 (PMC4506402; doi:10.1186/1756-8935-8-9)
Supplement: Supplementary file 10 — Additional file 10: Cholesterol biosynthesis pathway. (PDF 1 MB) [file 13072_2014_359_MOESM10_ESM.pdf]

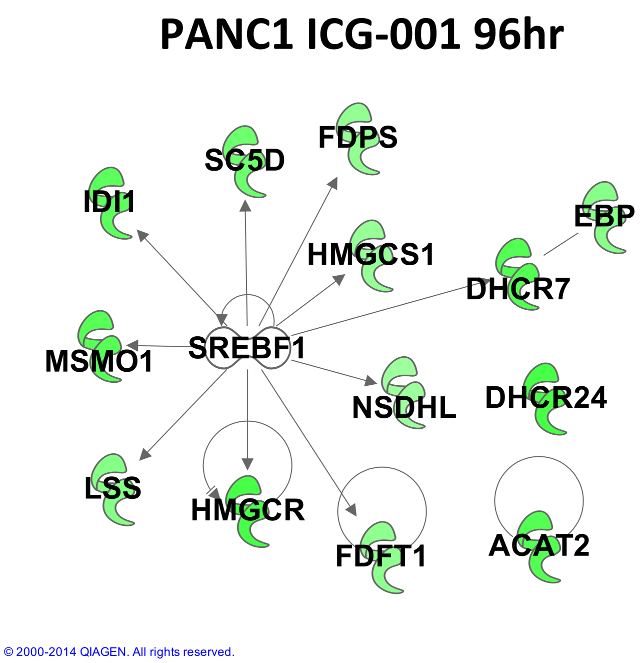

Fold change

Symbol	ICG-001 96hr	C646 96hr
ACAT2	-2.0	1.0
DHCR24	-2.1	1.3
DHCR7	-2.0	1.4
EBP	-1.4	1.6
FDFT1	-1.3	1.5
FDPS	-1.3	1.4
HMGCR	-2.1	1.2
HMGCS1	-1.2	1.4
IDI1	-1.9	1.2
LSS	-1.4	1.1
MSMO1	-1.9	1.3
NSDHL	-1.1	1.4

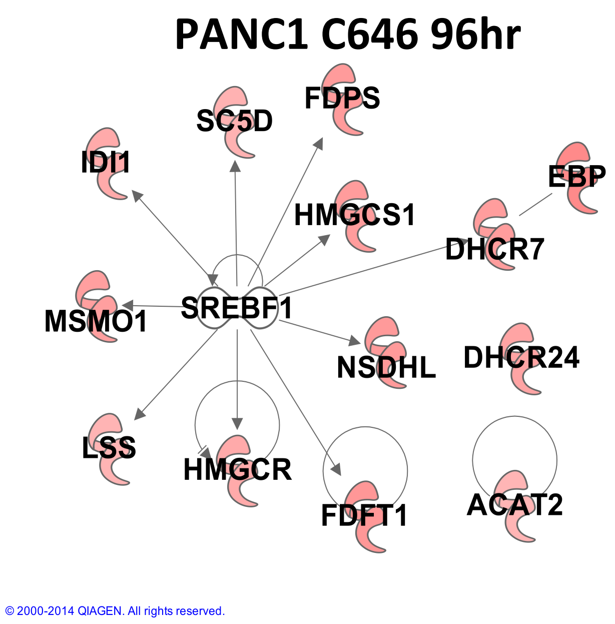

Additional File 10: ICG-001 negatively regulates the cholesterol biosynthesis network. IPA was used to show the relationships between SREBF1 and other genes involved in cholesterol biosynthesis that are affected by treatment of PANC1 cells with ICG-001 or C646. The arrows indicate direction interactions between the SREBF1 transcription factor and the other genes. Each of the indicated genes was down-regulated (indicated by the green color) by ICG-001 but up-regulated or unaffected by C646 (indicated by the red color); fold change for each gene is shown in the table.
